# Identification of an ortholog of the eukaryotic RNA polymerase III subunit RPC34 in Crenarchaeota and Thaumarchaeota suggests specialization of RNA polymerases for coding and non-coding RNAs in Archaea

**DOI:** 10.1186/1745-6150-4-39

**Published:** 2009-10-14

**Authors:** Fabian Blombach, Kira S Makarova, Jeannette Marrero, Bettina Siebers, Eugene V Koonin, John van der Oost

**Affiliations:** 1 Laboratory of Microbiology, Wageningen University, Wageningen, The Netherlands; 2 National Center for Biotechnology Information, NLM, National Institutes of Health, Bethesda, Maryland 20894, USA; 3 University Duisburg-Essen, Faculty of Chemistry, Biofilm Centre, Molecular Enzyme Technology and Biochemistry, Lotharstrasse, Duisburg, Germany

## Abstract

One of the hallmarks of eukaryotic information processing is the co-existence of 3 distinct, multi-subunit RNA polymerase complexes that are dedicated to the transcription of specific classes of coding or non-coding RNAs. Archaea encode only one RNA polymerase that resembles the eukaryotic RNA polymerase II with respect to the subunit composition. Here we identify archaeal orthologs of the eukaryotic RNA polymerase III subunit RPC34. Genome context analysis supports a function of this archaeal protein in the transcription of non-coding RNAs. These findings suggest that functional separation of RNA polymerases for protein-coding genes and non-coding RNAs might predate the origin of the Eukaryotes.

Reviewers: This article was reviewed by Andrei Osterman and Patrick Forterre (nominated by Purificación López-García)

## Findings

All Eukaryotes possess 3 distinct, multi-subunit RNA polymerases (RNAPs): RNA polymerase I (transcription of 16S and 23S rRNA), RNAP II (transcription of protein-coding mRNAs), and RNAP III (transcription of 5S rRNA, tRNA and some other small non-coding RNAs). Plants have two additional RNAPs involved in the transcription of small interfering RNA [1].

RNAP III has counterparts (either identical or paralogous) to all subunits of RNAP I and RNAP II [2]. In addition, RNAP III possesses the loosely bound RPC82/RPC34/RPC31 sub-complex. This sub-complex is present in all Eukaryotes, although RPC31 is missing in two major eukaryotic lineages (Alveolates and Excavates) [3]. Transcription initiation by RNAP III requires, among others, the TBP and TFIIIB70 proteins. TBP is shared with RNAP II, and the N-terminal region of TFIIIB70 is homologous to the RNAP II factor TFIIB, whereas the C-terminal region is specific for TFIIIB70. The archaeal RNAP (aRNAP) resembles RNAP II in its subunit composition [2]. Furthermore, the aRNAP machinery employs the transcription initiation factors TBP and TFB, which are orthologs and functional counterparts to the eukaryotic TBP and TFIIB/TFIIIB70, respectively [4].

In the RNAP II and aRNAP machineries, TFIIB and TFB are thought to recruit the RNAP directly to the transcription pre-initiation complex. In contrast, RNAP III requires the RPC34 subunit to mediate the interaction between TFIIIB70 and RNAP III [5-7]. Both the conserved N-terminal region and the unique C-terminal region of TFIIIB70 contribute to RPC34 binding [6,8]. Given the conservation of RPC34 in all eukaryotes and its central role in the recruitment of RNAP III to the pre-initiation complex, it seems likely that RPC34 played an important role in the evolution of the RNAP III transcription system. To address this possibility, we set out to identify potential archaeal homologs of RPC34.

## Identification of archaeal homologs of RPC34

Using PSI-BLAST search [9] (against the RefSeq database [10], with default parameters) with human RPC34 as the query (GI: 149640989), we detected a hit to a *Cenarchaeum symbiosum *(strain A) protein [GI: 118575757 with E-value = 5 × 10^-5^] after the first iteration. Reciprocal search starting from the *Cenarchaeum symbiosum *sequence [GI: 118575757; 244-362 aa] identified the first eukaryotic RPC34 homolog [GI:157138209 with E-value = 4 × 10^-10^] after the first iteration. All archaeal orthologs can be retrieved after the first iteration in the course of the same search (the complete information is available in Additional File 1). We identified apparent orthologs of RPC34 in all crenarchaeal and thaumarchaeal genomes as well as in several lineages of Euryarchaeota but not in Candidatus *Korarchaeum cryptofilum *OPF8, the only Korarchaeote sequenced so far (Additional File 1). None of these archaeal sequences are annotated as RPC34 homologs in the Refseq database. In agreement with the PSI-BLAST results, a Conserved Domain Database search [11] with various crenarchaeal and thaumoarchaeal sequences as queries identifies the statistically significant similarity (E-value ~0.001) of their C-terminal domain to a profile pfam05158, RNA polymerase RPC34 subunit. A similar result was obtained using HHPRED search [12]. For the same *Cenarchaeum symbiosum *A query, pfam05158 (RNA polymerase Rpc34 subunit) was detected with E-value = 6.6 × 10^-23^; in the same HHPRED search, the sequence corresponding to the structure of human RPC34 winged helix-turn-helix (wHTH) domain [PDB:2dk5] was detected with E-value = 2 × 10^-11^. The next most similar family of wHTH-domain-containing proteins was the MarR family of transcriptional regulators (pfam010470, with E-value = 1.2 × 10^-9^). The latter observation is also consistent with the PSI-BLAST search results of the HTH region of archaeal RPC34 orthologs in which MarR family sequences were identified as the closest hits. Most likely, this relationship between RPC34 and MarR is the cause of the misannotation of some of the apparent archaeal orthologs of RPC34 as MarR family transcriptional regulators [e.g. GI:18313992]. Thus, the N-terminal region of archaeal RPC34 orthologs contains a wHTH domain, whereas the C-terminal domain is a distinct Zn-finger domain shared with most eukaryotic RPC34 sequences.

**Figure 1:**
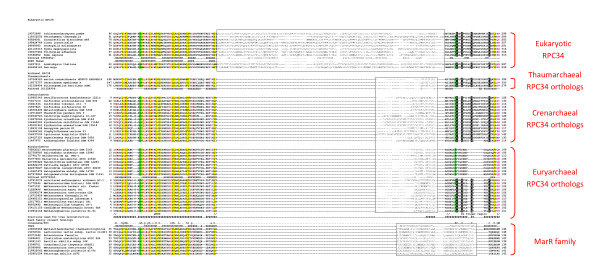
**The archaeal RPC34 orthologs - Multiple sequence alignment of eukaryotic RPC34 proteins and their archaeal orthologs**. The sequences are denoted by their GI numbers and species names. The positions of the first and the last residues of the aligned region in the corresponding protein are indicated for each sequence. The semi-transparent rectangles show regions that are aligned only in the respective groups of sequences. The cysteine residues comprising the Zn-finger motif are shown in reverse shading. Secondary structure predictions are shown underneath the respective groups of sequences. Secondary structure derived from the crystal structures of human RPC34 wHTH domain and a MarR family protein is also shown; 'H' indicates α-helix, 'E' indicates extended conformation (β-strand). The coloring is based on the consensus shown underneath the alignment of the RPC34 family; 'h' indicates hydrophobic residues (ACFGILMVWY), 'p' indicates polar residues (CDEHKNQR), 'a' indicates aromatic residues (WFYH), "s" indicated small residues (ACDGNPSTV).

The multiple alignment of the eukaryotic RPC34 sequences and their archaeal orthologs reveals conservation of two regions (Figure 1). In agreement with the above observations, the first region corresponds to the N-terminal wHTH domain (with all structural elements of wHTH, namely, three α-helices and two β-strands, preserved) whereas the second conserved region corresponds to the Zn-finger domain with the unique CxxC-x(3-5)-C-x(4-10)-C signature. There are substantial differences in the Zn-finger domain architectures of the euryarchaeal domains, on the one hand, and the crenarchaeal, thaumarchaeal and eukaryotic domains, on the other hand. In particular, the Zn-finger signature cysteines are not conserved in all sequences from Halobacteriales. All eukaryotic sequences contain a structured insert between the wHTH and the Zn-finger domains (according to PSIPRED [13] secondary structure prediction) that probably represents a distinct domain. Thaumoarchaeal proteins contain an extended region of low complexity N-terminal of the wHTH domain.

## Phylogenetic analysis of the RPC34 family

**Figure 2:**
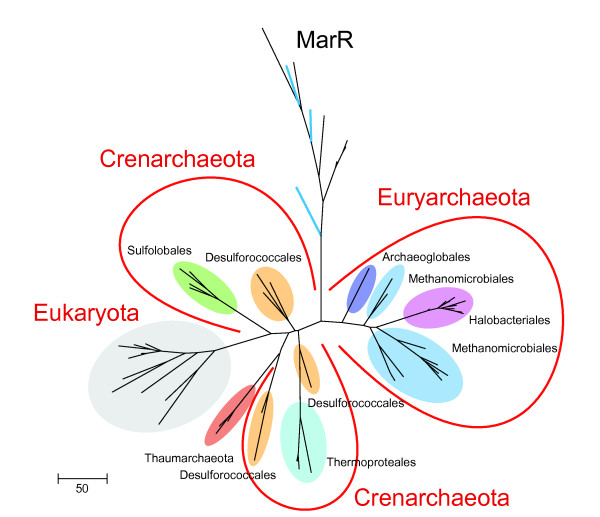
**The archaeal RPC34 orthologs - Phylogenetic analysis of the RPC34 family**. The ML tree was rooted using selected representatives of the MarR family as the outgroup (archaeal members of this group are shown in blue). A version of the tree with complete information for all the sequences used for tree construction and RELL bootstrap values is available in Additional File 2.

We constructed a phylogenetic tree from the alignment of the wHTH and Zn-finger domains of the eukaryotic and archaeal RPC34 orthologs, using the MarR family wHTH domain as an outgroup (Figure 2). Consistent with the apparent synapomorphies in the Zn-finger domain architecture (see above), the phylogenetic analysis shows that eukaryotic proteins group with crenarchaeal and thaumarchaeal sequences with reliable bootstrap support, excluding all euryarchaeal sequences (Figure 2 and Additional file 2). Moreover, the eukaryotic lineage is rooted deeply within the crenarchaeal-thaumarchaeal subtree (Figure 2), suggesting that eukaryotic RPC34 indeed originates from an ancestor that belonged to this group of archaeal proteins.

## Analysis of neighborhood of archaeal RPC34 orthologs

**Figure 3:**
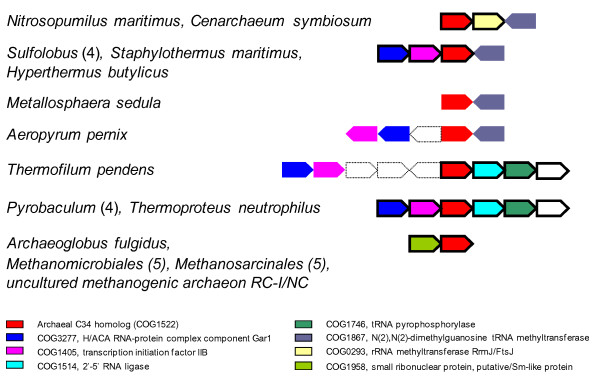
**The archaeal RPC34 orthologs - Genome context analysis of archaeal RPC34 orthologs**. Bold lines indicate possible co-transcription (intergenic region < 100 bp). The genome of *Sulfolobus solfataricus *P2 contains a Gar1 homolog (LocusTaq Sso6830), which is not annotated as a gene, upstream of Sso0944.

To gain insight into possible functions of the archaeal RPC34 orthologs, we analyzed the genomic context of the respective genes. In thaumarchaeal and crenarchaeal genomes, the RPC34 genes co-localize and are predicted to be co-transcribed with several genes for proteins involved in modification or processing of tRNA and rRNA (Figure 3). In the majority of crenarchaea, the RPC34 gene is also potentially co-transcribed with a gene for a TFB paralog (COG1405). Generally, archaeal genomes encode at least two TFB paralogs, so an intriguing possibility is that the crenarchaeal RPC34 ortholog interacts with a specific TFB paralog analogously to the interaction of eukaryotic RPC34 with the TFIIB paralog TFIIIB70. Genes encoding the euryarchaeal RPC34 orthologs, with the exception of those from Halobacteriales, are predicted to be co-transcribed with genes for Sm-like protein paralogs (COG1958). The *Archaeoglobus fulgidus *homolog Af-Sm2 has been shown to co-immunoprecipitate with RNase P RNA, and Sm-like proteins are generally believed to form ribonucleoprotein complexes [14].

## Possible role of the archaeal RPC34 ortholog in transcription

The genomic context of the archaeal RPC34 ortholog as well as the analogy with the eukaryotic RPC34, suggest that these archaeal proteins might be involved in transcription of rRNA and tRNA genes. It has been shown that, in a reconstituted *in vitro *transcription system from *Sulfolobus shibatae *transcription from rRNA and tRNA promoters could be successfully initiated in the absence of the RPC34 ortholog [15]. Hence, there is no strict RPC34 requirement for recognition of these promoters and recruitment of the aRNAP. Nevertheless, a regulatory role of this protein in the transcription of structural RNAs by aRNAP appears likely. The wHTH motif might mediate protein-DNA-interactions given that the eukaryotic RPC34 was cross-linked to DNA in transcription initiation complexes [16] but, to our knowledge, RPC34 has not been reported to contribute to promoter recognition. Electron microscopy revealed the position of the RPC82/RPC34/RPC31 sub-complex in the core RNAP III close to the "clamp" formed by the N-terminal part of the largest subunit, C1 [17]. The "clamp"-domain is conserved in all multi-subunit RNAPs, but an RNAP III-specific region is thought to be important for RPC34 binding specificity [3]. The archaeal RPC34 ortholog might similarly recruit aRNAP to the transcription pre-initiation complex via the "clamp"-domain and so enhance the transcription of structural RNAs.

## On the origin of eukaryotic RNAP multiplicity

The detection of a RPC34 ortholog in Archaea suggests that the separation of RNA polymerases into dedicated forms for the transcription of protein-coding genes and genes for structural RNAs (eukaryotic RNAP II and RNAP III, respectively) might have evolved already in Archaea and was inherited by Eukaryotes from the "archaeal parent". In this scenario, the archaeal RPC34 ortholog would modulate the specificity of the single aRNAP, whereas in Eukaryotes the specialization deepened as a result of the duplication of the genes coding for other RNAP subunits and general transcription factors. Experimental analysis of the functions of the archaeal RPC34 ortholog will provide a direct test of this hypothesis.

The nature of the archaeal "parent" of eukaryotes is a wide open question [18,19]. Detailed comparison of individual functional systems allows partial reconstruction of the gene repertoire of this elusive entity. With respect to the transcription system, the present findings add to the other recent observations that reveal the existence of RNAP subunits and transcription factors that are specifically shared between eukaryotes and Crenarchaeota, along with either Thaumarchaeota or Korarchaeota [20-23].

## Methods

### Sequence analysis

Refseq database at the NCBI [10] was used for PSI-BLAST searches. Database searches were performed using PSI-BLAST [9] with default. We also used the remote homology identification servers for CDD-search [11] and HH search [12]. Multiple alignments of protein sequences were constructed by using MUSCLE program [24], followed by a minimal manual correction on the basis of local alignments obtained using PSI-BLAST [9]. Protein secondary structure was predicted using the PSIPRED program [13].

Maximum likelihood (ML) phylogenetic trees were constructed from the alignment of  archaeal RPC34 orthologs (the positions used for reconstruction are shown in Figure 1), by using the MOLPHY program [25] with the JTT substitution matrix to perform local rearrangement of an original Fitch tree [26]. The MOLPHY program was also used to compute RELL bootstrap values.

## Abbreviations

RNAP: RNA polymerase; aRNAP: archaeal RNA polymerase; wHTH: winged Helix-turn-helix.

## Competing interests

The authors declare that they have no competing interests.

## Authors' contributions

FB, JM, and KM performed sequence analysis. FB, KM, and EK wrote the initial draft of the manuscript. JM, BS, and JO wrote the final manuscript. BS, EK, and JO coordinated the study. All authors read and approved the final version of the manuscript.

## Reviewers' comments

### Reviewer's report 1

#### Andrei Osterman, Burnham Institute

A compact and insightful article of F. Blombach et al. proposes and provides a compelling genomic evidence for a very interesting evolutionary hypothesis shedding new light on the origin of the eukaryotic transcription machinery. Based on detailed comparative analysis of sequences, domain organization and genome context, the authors predicted a role of an uncharacterized archaeal protein in the transcription of noncoding RNAs, analogous to RPC34 subunit of the eukaryotic RNAPIII. In addition to important evolutionary implications, this bioinformatic analysis yielded a testable functional assignment that should and, due to this publication, most likely would soon be challenged by focused experiments.

Andrei Osterman

### Reviewer's report 2

#### Patrick Forterre, Université Paris-Sud/Institut Pasteur (nominated by Purificación López-García, Université Paris-Sud)

The paper by Blombach and colleagues describes the discovery, using *in silico *methods, of an archaeal homologue of the eukaryotic RNA polymérase III subunit RPC34. This is a very interesting finding, since Archaea contain otherwise a single RNA polymerase which harbours subunits homologous to those of eukaryal RNA polymérase II. Genome context analysis suggests that the archaeal RPC34 homologue is involved in RNA metabolism. Very interestingly, the authors notice that the gene encoding the archaeal RPC34 is often potentially co-transcribed with a TFB paralogue. The authors suggest that some specialization occurs in Archaea between transcription of protein coding genes and non-coding genes (tRNA and/or rRNA genes). Both type of transcription being driven by different TFB, the TFB required for the transcription of non-coding genes interacting with RPC34. It is known that transcription of tRNA or rRNA genes *in vitro *by archaeal RNA polymerase can occur in the absence of this protein. It will be nevertheless interesting to test this hypothesis by checking the effect of this protein on such system, for example in competition experiment with different types of promoter and different TBP. It will be also important to test the function of these proteins in vivo using the genetic systems recently developed for *Sulfolobus *species. The publication of this nice short paper will certainly encourage different labs to perform this kind of experiments. On the other hand, one cannot exclude the possibility that the archaeal RPC34 and related TBP are not involved in transcription per se but play another fundamental role in archaeal RNA metabolism. Interestingly, the archaeal RPC34 homologue is present in crenarchaea and thaumarchaea, but not in korarchaea and euryarchaea, the latter containing a more distantly related homologue. This indicates that the RPC34 was present in the last common archaeal ancestor and was later on lost in euryarchaea. The authors suggest from their data that the specialization of RNA polymerase between those transcribing coding and non-coding genes might have evolved already in Archaea and was inherited by Eukaryotes from the "archaeal parents". I previously criticized this notion of archaeal parents, noticing that we don't descend from Apes. Eugene Koonin correctly pointed out that I was wrong since we are apes indeed! However, are Eukaryotes Archaea? We don't know. It might be that Archaea are reduced proto-eukaryotes? In my opinion, it's still a prejudice to consider that Eukarya evolved from Archaea. I would say that the data presented in this nice paper indicate that the RPC34 protein was present in the last common ancestor of Archaea and Eukarya. As an alternative to the hypothesis proposed by the authors, it could be that this ancestor contained the ancestor of RNA polymerase III and that this protein (but not RPC34) was lost in Archaea (streamlining).

***Authors' response***: *We appreciate the constructive remarks and would like to briefly comment on only two aspects. First, it is hard to agree that "one cannot exclude the possibility that the archaeal RPC34 and related TBP are not involved in transcription per se but play another fundamental role in archaeal RNA metabolism". All we know about these proteins points to direct involvement in transcription, so this seems to be a safe bet. Of course, our suggestion that they are involved specifically in structural RNA synthesis is far more speculative. Second, about the "archaeal parent" of eukaryotes, very briefly, because this issue is far beyond the scope of the paper. Although much of it is semantics, meaningful distinctions can be made. Humans are indeed apes, the third species of chimpanzee by any legitimate criterion used in evolutionary biology. By contrast, eukaryotes are not archaea for the crucial reason that their genetic makeup is an archaeo-bacterial chimera. This is the reason why we find it preferable to speak of the archaeal "parent" of eukaryotes rather than the archaeal ancestor. This logic is not much affected by the exact nature of the archaeal parent - whether it was a typical archaeon or a derived one with some evolved eukaryotic features*.

## Supplementary Material

Additional file 1 **The list of all identified RPC34 representatives in archaeal genomes, selected eukaryotic orthologs and selected MarR-family wHTH transcriptional regulators**. The data provided represent list of all identified RPC34 representatives in archaeal genomes proteins and other proteins that were further analyzed in this work. Click here for file

Additional file 2 **The phylogenetic tree in rectangular format**. The phylogenetic tree in rectangular format with bootstrap values and full names of organisms used for tree reconstruction. Click here for file
