# Supplementary figures and images for: Identification of an ortholog of the eukaryotic RNA polymerase III subunit RPC34 in Crenarchaeota and Thaumarchaeota suggests specialization of RNA polymerases for coding and non-coding RNAs in Archaea
Source: Biol Direct. 2009 Oct 14;4:39. doi: 10.1186/1745-6150-4-39 (PMC2770514; doi:10.1186/1745-6150-4-39)

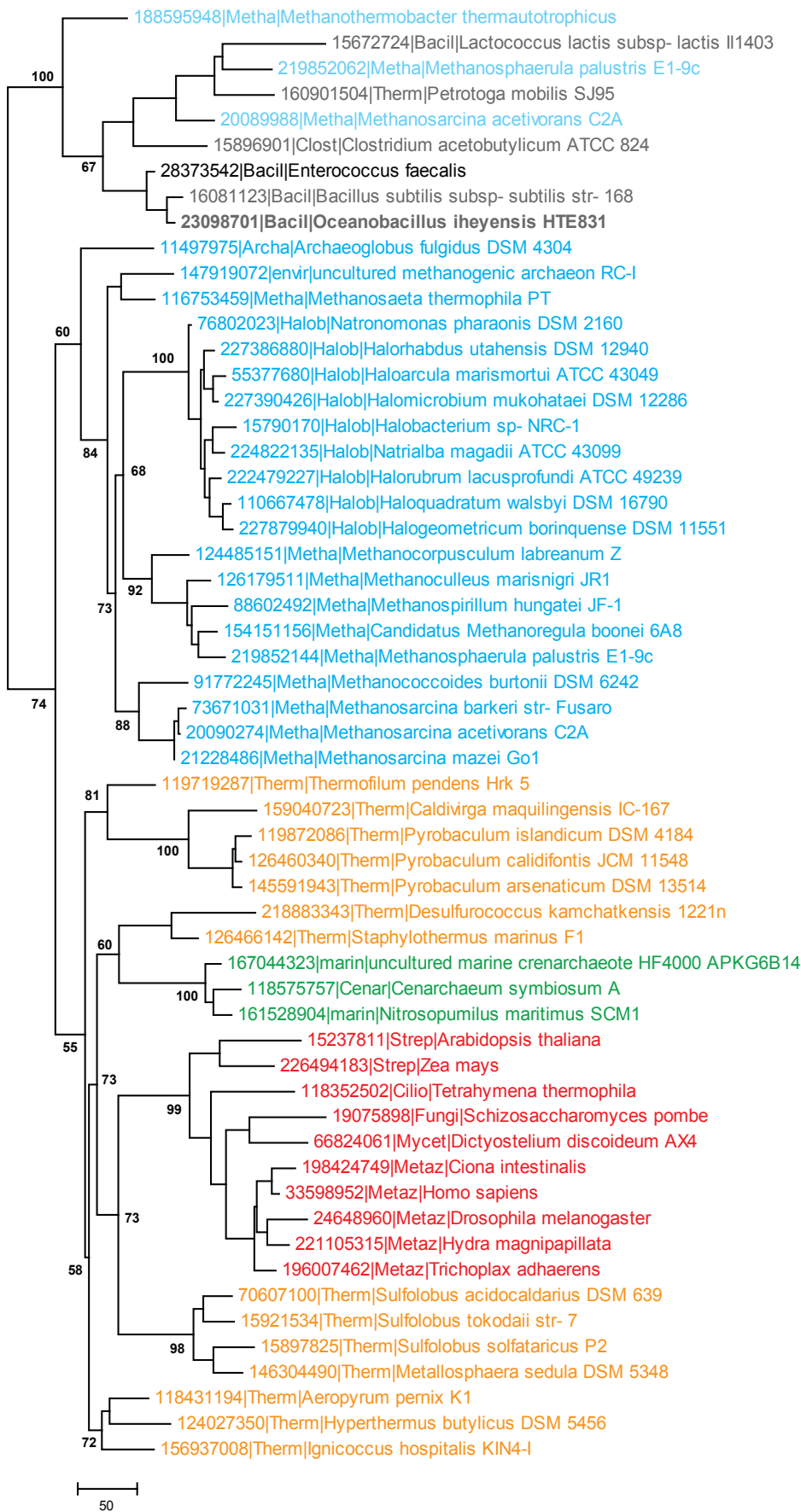

MarR

Euryarchaeota

Crenarchaeota

Thaumarchaeota

Eukaryota

Crenarchaeota

Supplement: Additional file 2 — The phylogenetic tree in rectangular format. The phylogenetic tree in rectangular format with bootstrap values and full names of organisms used for tree reconstruction. [file 1745-6150-4-39-S2.PDF]
